# Cell-based therapies have disease-modifying effects on osteoarthritis in animal models. A systematic review by the ESSKA Orthobiologic Initiative. Part 1: adipose tissue-derived cell-based injectable therapies

**DOI:** 10.1007/s00167-022-07063-7

**Published:** 2022-09-14

**Authors:** Carlotta Perucca Orfei, Angelo Boffa, Yosef Sourugeon, Lior Laver, Jérémy Magalon, Mikel Sánchez, Thomas Tischer, Giuseppe Filardo, Laura de Girolamo

**Affiliations:** 1 IRCCS Istituto Ortopedico Galeazzi, Laboratorio di Biotecnologie Applicate all’Ortopedia, Milan, Italy; 2 Applied and Translational Research Center, IRCCS Istituto Ortopedico Rizzoli, Bologna, Italy; 3 Rambam Health Care Campus, Haifa, Israel; 4 Department of Orthopaedics, Hillel Yaffe Medical Center (HYMC), Hadera, Israel; 5 Arthrosport Clinic, Tel-Aviv, Israel; 6 Technion University Hospital (Israel Institute of Technology) - Rappaport Faculty of Medicine, Haifa, Israel; 7 Cell Therapy Laboratory, Hôpital De La Conception, AP-HM, Marseille, France; 8 INSERM, NRA, C2VN, Aix Marseille Univ, Marseille, France; 9 SAS Remedex, Marseille, France; 10 Arthroscopic Surgery Unit, Hospital Vithas Vitoria, Vitoria-Gasteiz, Spain; 11 Advanced Biological Therapy Unit, Hospital Vithas Vitoria, Vitoria-Gasteiz, Spain; 12 Department of Orthopaedic Surgery, University of Rostock, Rostock, Germany; 13 Service of Orthopaedics and Traumatology, Department of Surgery, EOC, Lugano, Switzerland; 14 Faculty of Biomedical Sciences, Università Della Svizzera Italiana, Lugano, Switzerland

**Keywords:** Mesenchymal stromal cells (MSCs), Stem cells, Adipose tissue, Intra-articular, Injection, Disease-modifying, Osteoarthritis, Cartilage

## Abstract

**Purpose:**

The aim of this systematic review was to determine if adipose tissue-derived cell-based injectable therapies can induce disease-modifying effects in joints affected by osteoarthritis (OA).

**Methods:**

A systematic review was performed on three electronic databases (PubMed, Web of Science, Embase) according to PRISMA guidelines. A synthesis of the results was performed investigating disease-modifying effects in preclinical studies comparing injectable adipose-derived products with OA controls or other products, different formulations or injection intervals, and the combination with other products. The risk of bias was assessed according to the SYRCLE’s tool.

**Results:**

Seventy-one studies were included (2,086 animals) with an increasing publication trend over time. Expanded cells were used in 65 studies, 3 studies applied point of care products, and 3 studies investigated both approaches. Overall, 48 out of 51 studies (94%) reported better results with adipose-derived products compared to OA controls, with positive findings in 17 out of 20 studies (85%) in macroscopic, in 37 out of 40 studies (93%) in histological, and in 22 out of 23 studies (96%)  in immunohistochemical evaluations. Clinical and biomarker evaluations showed positive results in 14 studies out of 18 (78%) and 12 studies out of 14 (86%), while only 9 studies out of 17 (53%) of the imaging evaluations were able to detect differences versus controls. The risk of bias was low in 38% of items, unclear in 51%, and high in (11%).

**Conclusion:**

The current preclinical models document consistent evidence of disease-modifying effects of adipose-derived cell-based therapies for the treatment of OA. The high heterogeneity of the published studies highlights the need for further targeted research to provide recommendations on the optimal methodologies for a more effective application of these injective therapies for the treatment of OA in clinical practice.

**Level of evidence:**

II.

**Graphical Abstract:**

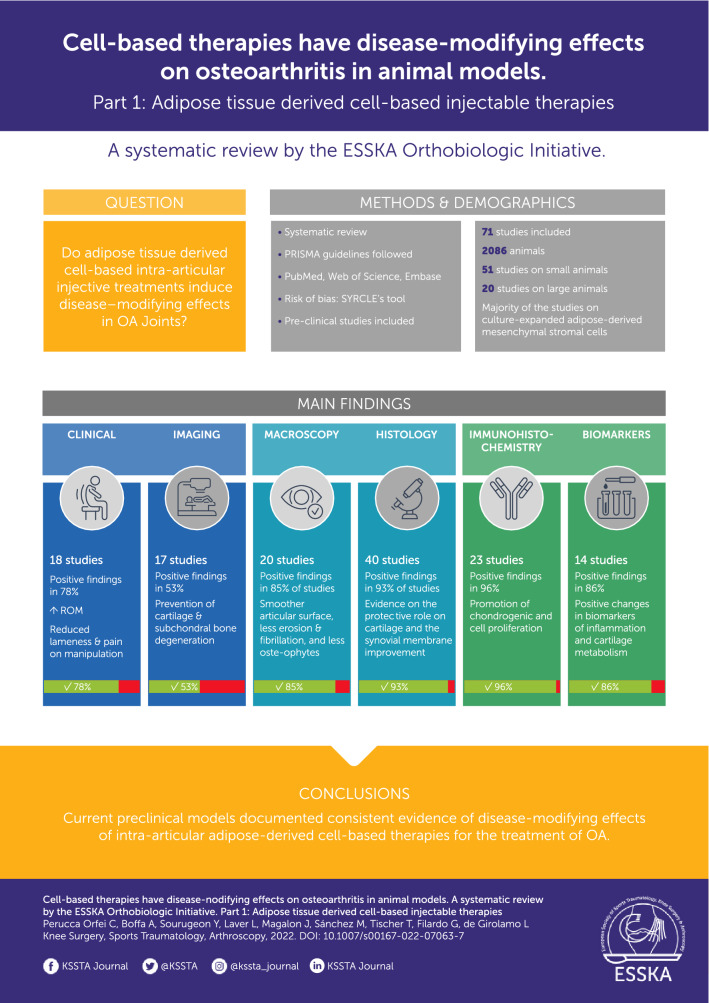

**Supplementary Information:**

The online version contains supplementary material available at 10.1007/s00167-022-07063-7.

## Introduction

Osteoarthritis (OA) is the most frequent joint disease in the world population [1]. Highly disabling, OA is characterized by both inflammatory and degenerative events that culminate in progressive cartilage damage and exposure of subchondral bone [2], eventually leading to pain and disability [1]. To date, there is no conservative therapy capable of curing this pathology or arresting its progression, with current interventions providing only temporary symptom relief. Recently, autologous biological injective therapies with anti-inflammatory, restorative, and regenerative properties have been introduced as promising strategy to treat OA. These products, namely orthobiologics, are blood-derived or cell-derived products that can be prepared from patient’s tissues either at the point of care or in authorized facilities using more complex laboratory procedures [3].

The assumption underlying the use of cell-based orthobiologics is closely related to the properties and functions of the cell type used, which must have the ability to determine a specific action at the target site [4]. From this perspective, mesenchymal stromal cells (MSCs) are ideal candidates for the treatment of damaged tissues given their restorative and pro-regenerative properties [5]. These cells can be found in most of the vascularized tissues, being a subtype of pericytes with pro-regenerative properties, and in particular in adipose tissue, bone marrow, and foetal annexes [6–8]. The former is not only abundant and very easy to harvest through a simple liposuction procedure, but it also contains one of the best performing MSC populations [9]. The key therapeutic effector of adipose-based orthobiologic products is the stromal vascular fraction (SVF) that, together with precursor and mature endothelial cells, pericytes, lymphocytes, pre-adipocytes and mature adipocytes, also contains adipose-derived MSCs (ASCs) [9]. Abundant literature has demonstrated the anti-inflammatory and adaptive properties of ASCs based on environmental conditions, with anti-inflammatory effects on chondrocytes and synoviocytes as well as polarization of M0 macrophages and dendritic cells towards anti-inflammatory phenotypes [10–12]. Given these premises, from a clinical perspective, the autologous use of ASCs would be expected not only to counteract inflammatory processes, but also to promote regenerative processes in the joint synovium and the chondral surfaces [13], and therefore to exert disease-modifying effects in joints, as already demonstrated in blood-derived products in preclinical studies [14].

Therefore, the aim of this systematic review was to investigate in preclinical studies the presence of disease-modifying effects driven by cell-based products used for the injective treatment of OA. The usefulness of this systematic review relies on the possibility to get pivotal information on the disease-modifying effects of adipose-based orthobiologics for the treatment of OA which is otherwise impossible to collect in a clinical setting due to ethical and practical concerns. While additional publications by this group (The ESSKA Orthobiologic Initiative = ORBIT) will investigate other MSC sources, this article will focus on the effects of adipose tissue derived products. This systematic review will help to define the advantages of using orthobiologics for the treatment of OA, in order to achieve a consolidated background for using these therapies in the daily clinical practice in the near future.

## Materials and methods

### Search strategy and article selection

A systematic review of the literature was performed according to the Preferred Reporting Items for Systematic Reviews and Meta-Analyses (PRISMA) guidelines on the intra-articular use of MSCs to address joints affected by OA. The search was conducted on January 10, 2022 on three electronic databases (PubMed, Web of Science, and Embase) with no time limitation and without any filters, using the following string on titles and abstracts: (MSC OR mesenchymal cell OR stem cell OR stromal cell OR progenitor cell OR bone marrow concentrate OR bone marrow aspirate concentrate OR BMAC OR micro-fra* adipose tissue OR microfra* adipose tissue OR stromal vascular fraction OR SVF OR amniotic suspension allograft OR ASA OR placenta* OR umbilical cord OR amnio*) AND (osteoarthritis). The screening process and analysis were conducted by two independent authors (CP and YS) and any discrepancies between them were resolved by discussion and consensus with a third author (AB).

**Fig. 1:**
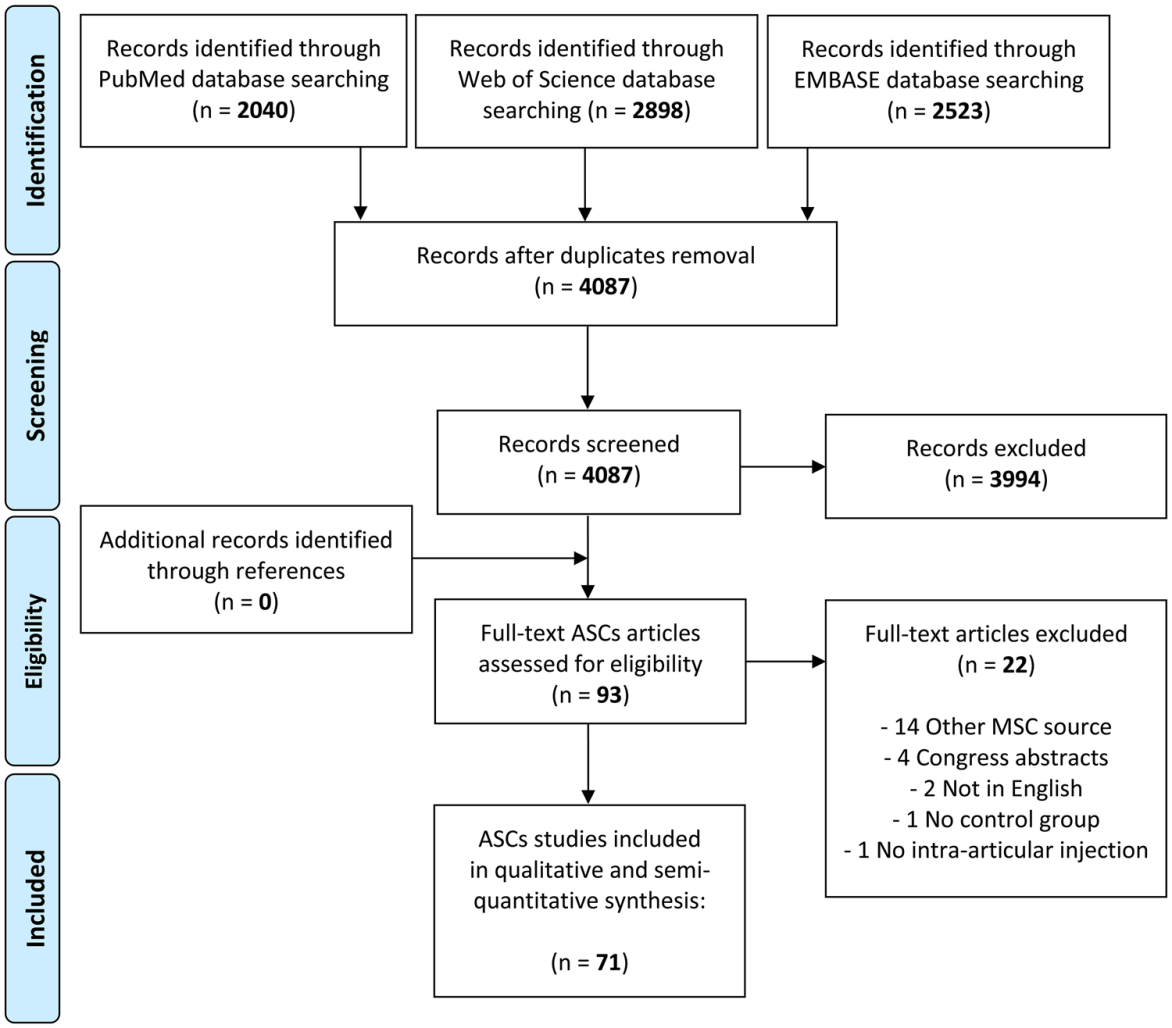
PRISMA flowchart of the study selection process. ASCs, adipose-derived mesenchymal stromal cells

First, the reviewers screened the resulting records by title and abstract, then the full texts of selected manuscripts were entirely screened according to the following inclusion criteria: animal studies, written in English, on cell therapy as purely injective treatment for cartilage degeneration and OA. Exclusion criteria were: in vitro or clinical studies, congress abstracts, literature reviews, articles written in other languages, studies on joint diseases different from OA, studies analyzing combined surgery (e.g., scaffolding procedure, arthroscopy), studies on the use of secretome and extracellular vesicles from MSCs, and studies reporting the use of MSCs without a control group or the combined use of MSCs with another product without analyzing the specific contribution of MSCs treatment. In addition, the reference lists from the selected papers and previously published relevant reviews were also screened. The flowchart reported in Fig. 1 graphically describes the systematic review process. The current manuscript focuses on adipose-derived products, while additional publications by this group (The Orthobiologic Initiative = ORBIT) will investigate other MSC sources separately, which were therefore excluded for this analysis.

### Data extraction and quality assessment

For the included studies, all relevant data were extracted and reviewed from article texts, tables, and figures, and then summarized and analyzed for the purpose of the present work. In particular, the following data were collected for each study: authors, journal and year of publication, number and type of evaluated animals, involved joint, OA model, type of treatments, follow-up length, results, and adipose-based orthobiologic products characteristics, including source, origin, dose, additional procedures, injective protocol, and processing modality (expanded versus “point of care”). A synthesis of the obtained results was performed analyzing the disease-modifying effects of intra-articular application of the different preparations, as assessed by objective evidence measures of effect (imaging, macroscopic, histological, or immunohistochemical) on OA processes going beyond the mere symptomatic improvement. The clinical outcome was reported as well. This was achieved by evaluating studies that compared animals treated with adipose-based orthobiologic products and OA controls (vehicle injection or no treatment). Moreover, other results were analyzed when available regarding the benefits provided by different doses or injection schedules, the effects versus other injectable treatments, and finally the effects derived from the combination of adipose-derived products with other products exploring potential synergistic effects.

The risk of bias of the included articles was assessed according to the Systematic Review Centre for Laboratory animal Experimentation (SYRCLE)’s tool [15]. This tool is an adapted version of the Cochrane Collaboration RoB Tool and contains 10 items related to the types of bias: selection bias, performance bias, detection bias, attrition bias, reporting bias, and “other” biases. All items could be judged as ‘yes’ (low risk of bias), ‘no’ (high risk of bias), and ‘unclear’ (unclear risk of bias). The assessment was independently performed by 2 authors (CP and YS), and any divergence was resolved through discussion and consensus with a third author (AB).

## Results

### Study selection and analysis

**Fig. 2:**
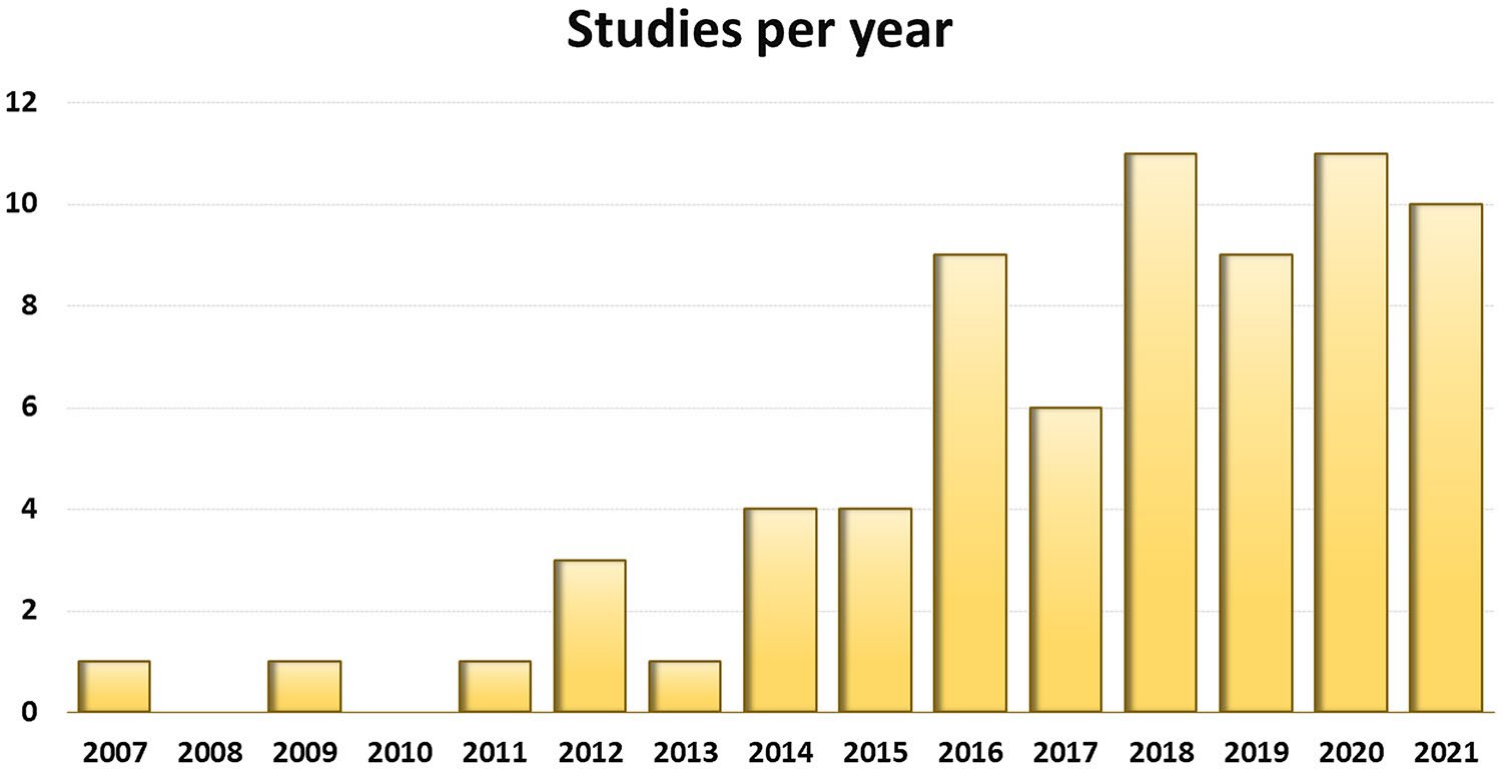
Animal studies on intra-articular adipose-derived MSCs injections to address OA over the years

A total of 4087 potential articles were identified according to the search strategy, resulting in 71 studies included in the qualitative data synthesis (Fig. 1). Since the first report in 2007, the publication trend remarkably increased over the years, with over 50% of articles published since 2018 (Fig. 2). Fifty-one studies were on small animals (32 on rodents, 19 on rabbits) and 20 studies on large animals (9 on dogs, 6 on sheep, 4 on horses, 1 on goats), for a total of 2086 animals, of which 955 rodents, 654 rabbits, 224 dogs, 112 horses, 111 sheep and 30 goats. The treated joints were knees in 61 articles, hips in 4 articles, ankles in 2 articles, elbows and temporo-mandibular joints in 1 article each, and 2 articles focussed on multiple joints. The OA model was surgically induced in 44 studies (mostly through meniscectomy and/or ligament transection), chemically induced in 18 studies (through the injection of chondrotoxic or pro-inflammatory products such as collagenase, mono-iodoacetate, papain, streptozotocin), naturally occurring in eight studies (veterinary studies), and induced by joint immobilization in 1 study.

Treatments were reported as allogeneic in 30 studies, autologous in 17 studies, and xenogeneic in 18 studies, with 16 of them using human-derived ASCs. Moreover, one study used both autologous and xenogeneic (human) ASCs [16], one study used both allogeneic and xenogeneic (human) ASCs [17], while four studies did not specify the MSC origin. Expanded ASCs were used in 65 studies, 3 studies used adipose-derived cell-based products produced at the point of care, and 3 studies applied both approaches. Twenty-four studies evaluated adipose-based orthobiologic products with additional procedures including microencapsulation, 3D cell culture dishes with spheroids, induction of overexpressed transcription factors, preconditioning with vitamin E, or chondrogenic differentiation. ASC dose was described in 70 studies and ranged from 2.0 × 10^4^ to 5.0 × 10^7^, while the amount of the injected volume was reported in 64 studies and ranged from 6.0 μL to 2.0 mL in small animals and from 0.5 mL to 5.0 mL in large animals. The most common injection frequency protocol was a single injection administration (59 studies), while multiple-injection protocols were used in 12 studies, with injection intervals ranging from 3-day to 4-week intervals. The follow-up ranged from 1 week to 12 months. Further details are reported in the supplementary material.

### Disease-modifying effects on OA joints

**Fig. 3:**
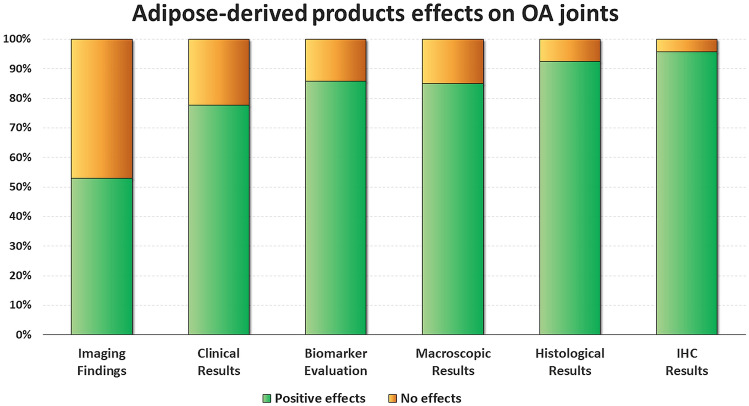
ASCs effects on OA joints. The bar chart shows the percentage of studies that met the specific effects. Positive effects vs no effects in imaging findings (*n* = 17), clinical results (*n* = 18), biomarker evaluation (*n* = 14), macroscopic results (*n* = 20), histological results (*n* = 40) and immunohistochemical results (*n* = 23). ASCs, adipose-derived mesenchymal stromal cells; IHC, immunohistochemistry; OA, osteoarthritis

Fifty-one studies (42 in small animals and 9 in large animals) investigated the disease-modifying effects of intra-articular adipose-derived injections in comparison to OA controls (untreated joints or vehicle injections), of which 46 reported the effects on cartilage and 10 the effects on synovial membrane. Overall, 48 studies (94.1%) reported better results in animals treated with adipose-derived products compared to OA controls and 3 studies (5.9%) revealed no improvement following injection. Noteworthy, no studies showed detrimental effects. In detail, 17 out of 20 studies (85.0%) with macroscopic evaluations (gross morphological scores) reported overall better results, 37 out of 40 studies (92.5%) with histological evaluations, and 22 out of 23 studies (95.7%) with immunohistochemical evaluations. Similar results were observed comparing small and large animal OA models (95.2% and 88.9% overall positive disease-modifying effects, respectively). A more detailed analysis is reported in the following paragraphs and in Fig. 3.

### Disease-modifying effects at the cartilage level

Out of the 46 studies investigating the disease-modifying effects on cartilage tissue, 44 (95.7%) reported positive results as a treatment for OA affected joints. In particular, at macroscopic evaluation of damaged cartilage areas, appearance was milder compared to OA controls, reporting a smoother articular surface with less erosion, fibrillation, and osteophytes [α]. Histological analysis provided evidence on the protective role on cartilage, featuring improved tissue thickness and arrangement of chondrocytes, accompanied by decreased extracellular matrix loss and higher amount of cartilage specific extracellular matrix (proteoglycan and type II collagen) [β]. Moreover, less severe sclerosis and lower thickening of the subchondral bone plate was observed [18]. Immunohistochemical analysis also demonstrated the promotion of chondrogenic (type II collagen and aggrecan) and cell proliferation (proliferating cell nuclear antigen—PCNA) marker expression, while reducing the expression of fibro-cartilaginous (type I collagen), catabolic (matrix metalloproteinase and aggrecanase), apoptotic (caspase 3), and inflammatory (tumour necrosis factor-α—TNF-α, nuclear factor kappa-light-chain-enhancer of activated B cells—NF-κB) markers [γ].

### Disease-modifying effects at the synovial membrane level

Only 10 studies investigated the disease-modifying effects of intra-articular adipose-derived products injections on the synovial membrane, with 6 of them (60%) reporting positive results and the others showing no differences compared to OA controls. In particular, adipose-derived products improved the synovitis status reducing the thickness of the lining layer of the synovial membrane and decreasing the infiltration of inflammatory cells (mononuclear cells and neutrophils) in the sub-synovium [δ]. Immunohistochemical analyses showed that ASCs treatment inhibited metalloproteinase-1 (MMP-1) and TNF-α expression, and reduced the ratio of inflammatory macrophages (iNOS positive-cells) in the synovial membrane [19, 20].

### Effects on OA biomarker profile

Fourteen studies evaluated the disease-modifying effects through the measurement of synovial fluid (SF) or serum biomarkers related to cartilage metabolism or inflammation. Among these, 12 studies (86%) reported positive changes with a decrease in the SF concentration of lymphocytes and inflammatory biomarkers, such as interleukin-1β (IL1-β), IL-6, TNF-α, and prostaglandin E2 (PGE2) [ε]. Moreover, they provided a reduction of SF matrix-degrading enzymes (MMP-3 and MMP-13) and C-terminal telopeptide of type II collagen (CTX2) [21, 22], as well as the serum levels of inflammatory markers, such as IL-6, TNFα, S100 Calcium Binding Protein A9 (S100A9), and monocyte chemoattractant protein 1 (MCP-1), the serum levels of cartilage oligomeric matrix protein (COMP), and those of osteoprotegerin (OPG) [ζ].

#### Clinical effects

Eighteen studies quantitatively evaluated the clinical effects of adipose-based orthobiologic products: 14 studies (78%) reported better clinical results compared to OA controls in both small (6 out of 7) and large animals (8 out of 11), with increased passive range of motion and reduced lameness and pain on manipulation [η]. The injections also promoted paw-withdrawal latency and threshold, as well as better weight bearing [θ].

#### Imaging analysis

Seventeen studies evaluated the effects by imaging analysis: nine studies (52.9%) reported significant differences in favour of adipose-derived products, while eight studies observed no differences. In particular, micro-computed tomography (micro-CT) evaluation was performed in six studies, with positive effects in five studies in terms of prevention of cartilage and subchondral bone degeneration and improvement of bone thickness and volume [23–27]. Radiographic evaluation was performed in 6 studies with only one study showing a statistically significant difference in favour of adipose-derived products [28]. Magnetic resonance imaging (MRI) was used in 4 studies with positive results observed in 2 studies in terms of improvement of the cartilage repair process with significantly higher Magnetic Resonance Observation of Cartilage Repair Tissue (MOCART) scores than in the untreated one [29, 30]. Moreover, positron emission tomography (PET) analysis performed in one study reported a significant difference compared to untreated OA joints in enhancing cartilage regeneration [31]. Finally, one study used back scattered electron imaging to assess the morphology, porosity, and heterogeneity of mineralization within the subchondral bone without observing significant differences between ASC-treated and untreated groups [32]

#### Comparison of formulations and protocols

Twenty-nine studies compared injective protocols involving ASCs or adipose-derived cell-based products, differing in origin, preparation, doses, times and interval of administration, and additional cell procedures/pre-treatments. In particular, 11 studies evaluated low-dose versus high-dose ASCs, reporting controversial results. On one hand, five studies showed a dose-dependent effect of the treatment, with high doses resulting in better macroscopic and histological results [ι]. On the other hand, five studies demonstrated comparable effects among different doses [κ]. Another study reported better cartilage preservation in the intermediate dose group (0.6 × 10^7^/100 μL) with respect to low (0.18 × 10^7^/100 μL) and high dose (1.8 × 10^7^/100 μL) groups in a goat OA model [33].

Five studies compared disease-modifying effects of cultured ASCs vs freshly isolated cell-based products without finding consistent results. In fact, while two studies reported that cultured ASCs yielded better results in terms of MRI measures and macroscopic findings [30, 31], one study revealed that micro-fragmented adipose tissue (MFAT) provided better synovitis reduction and articular cartilage status compared to SVF and expanded ASCs [34], and another study demonstrated better results in terms of macroscopic findings and decrease of SF TNF-a and IL-6 in the enzymatic SVF group compared to expanded ASCs [22]. Another study did not find histological differences between expanded ASCs, SVF, and MFAT [35].

Three studies evaluated the injection of microencapsulated ASCs aggregated into spheroids, all showing improved chondroprotective effects and pain relief compared to ASCs alone [36–38].

Two studies compared products injected at different time points from OA induction, both observing a lower cartilage degeneration in the early injection groups compared to the late injection groups [39, 40].

Two studies investigated the role of the ASCs chondrogenic differentiation before injection: one study reported better histological results of pre-differentiated ASCs compared to undifferentiated ones in a rat OA model [41], while the other study did not show any difference in a rabbit OA model [42].

Tang et al. compared ASCs obtained from different adipose tissue sources (subcutaneous fat versus visceral fat) reporting higher chondroprotective effects in the subcutaneous ASCs group [43].

Finally, seven studies evaluated benefits of different additional procedures, reporting better results compared to ASCs alone in case of pre-treatments aimed to induce overexpression of Fos member of the AP-1 family (Fra-1) or bone morphogenetic protein‑9 (BMP-9), platelet-derived growth factor (PDGF) or PDGF receptor-β transduction, interferon-γ (IFN-γ) or signal transducer and activator of transcription 3 (STAT3). No advantages were observed when SOX transduction was induced [λ].

### Comparison with other injectable products

Five studies compared ASCs versus expanded bone marrow MSCs (BMSCs): 4 studies did not show differences in terms of clinical improvement, macroscopic and histological findings, while one study reported better mechanical properties after BMSCs injection compared to ASCs [μ]. A further study compared enzymatic SVF with expanded BMSCs, reporting higher improvement in flexion and SF profile for the BMSC group [44]. Five studies compared adipose-based orthobiologic products and PRP, showing controversial findings. Two studies demonstrated better results for adipose cell-based products in terms of macroscopic and histological findings in a rat OA model and in terms of clinical improvement in a randomized controlled trial on dogs with naturally occurred OA [ν]. Conversely, three studies showed similar histological and immunohistochemical benefits between the two treatments in surgically induced OA models (mice, rats, and dogs) [ξ]. Two studies used viscosupplementation as treatment comparison. While one study did not demonstrate any difference in a rabbit knee OA model, one study in a temporo-mandibular rabbit OA model reported a higher cartilage protection in the cell therapy group, even though no significant differences were found in terms of cartilage thickness [32, 45]. One study compared ASCs (expanded or SVF) versus expanded amniotic epithelial stem cells (AECs) in a sheep OA model. Among the three treatments, SVF provided the best histological performance while ASCs resulted in the least improvement of the three [22]. Finally, one study compared ASCs with human umbilical cord Wharton’s jelly-derived MSCs (WJMSCs) in a rat OA model, reporting a higher type II collagen expression in the WJMSCs group, although no other differences were observed in terms of histological analysis, micro-computed tomography, and immunohistochemistry staining [24].

### Effects of combined treatments

Five studies investigated the combined use of adipose-based orthobiologic products and hyaluronic acid to address OA models. All these studies reported a synergistic effect of this combined approach with higher inhibiting effects on cartilage degeneration progression compared to viscosupplementation alone [ο]. The combined use of expanded ASCs and PRP revealed a synergistic effect in two OA models with higher extracellular matrix synthesis, chondrocyte proliferation, and anti-inflammatory effects [46, 47]. Adipose-derived products also showed synergistic effects in addressing OA progression when combined with shockwave therapy [23, 24], chondral cell suspension [48], carboxymethyl chitosan [45], xanthan gum [49], thrombospondin 2 [28], or acupotomy [50]

### Risk of bias assessment

**Fig. 4:**
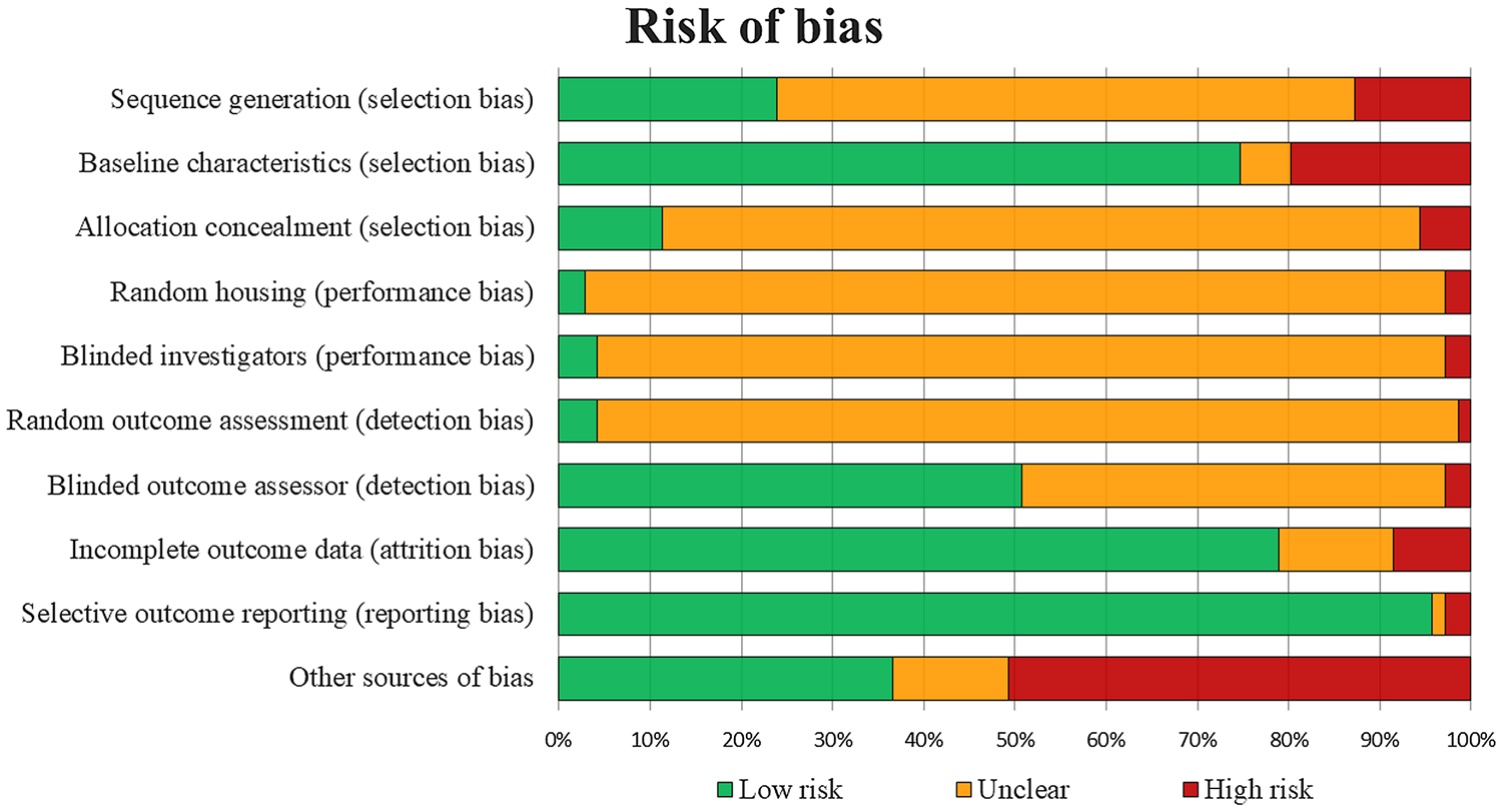
SYRCLE's risk of bias tool assessment of the included studies. The bar chart shows the percentage of all studies that met each quality item, scored as “Low risk”, “High risk”, or “Unclear”

There was a 79% agreement between the two authors involved in the evaluation of the risk of bias. Most items (51%) were rated as unclear, while low and high risk of bias were observed in 38% and 11%, respectively. The evaluation of risk of bias over time did not show a trend towards improving the quality of the included studies, with low-risk items reported in 37% vs 40% in more recent papers vs older ones. Details of the risk of bias assessment of all included studies are illustrated in Fig. 4.

## Discussion

This systematic review supports that injectable adipose-derived cell-based therapies exert disease-modifying effects on joint tissues in both small and large animal OA models. Among these effects, they provide a structural/macroscopic improvement of both cartilage and synovial membrane properties compared to untreated OA joints as well as a decrease in serum and SF levels of both inflammatory and cartilage degradation markers. These effects were also confirmed by improvement in clinical measures.

The high number of studies underlines the growing interest on this type of conservative treatment option for OA, confirmed by the increasing trend in publications covering this topic in recent years. Advantages of using adipose tissue derived cells and/or products are widely described in the literature, being even more advantageous than BMSCs in some respects, including higher cellular yield and remarkable regenerative and immunomodulatory potential [51–53]. A recent meta-analysis on the safety and efficacy of adipose tissue derived cell-based therapies in humans for the treatment of knee OA [54] showed a reduction in pain and an improvement in knee function at least up to two years [55]. Nevertheless, these therapies are still rather immature in the clinical setting for various reasons, including regulatory restrictions, heterogeneity of the techniques available, cost-effectiveness, and the lack of objective data concerning their effectiveness [56]. In the latter regard, most of the information on the biological effects of these injective therapies for treating OA resides in the preclinical setting. This systematic review assessed the disease-modifying effects of these treatments in animal studies to provide useful information regarding the clinical effectiveness observed in humans and to promote a consolidated background supporting the use of these procedures in daily clinical practice. The administration of adipose tissue derived cell-based injective therapies demonstrated to exert a protective role on the cartilage tissue at both histological and immunohistochemical level. These findings are largely supported by previous in vitro studies in which the trophic effects of MSCs (derived from fat or bone marrow) co-cultured with chondrocytes enhance cartilage formation, matrix deposition, and proliferative activity in chondrocytes [57].

In addition, data from these studies suggest that the injection of these products also exerts a positive effect on the synovial membrane, improving the synovitis [δ]. When co-cultured in vitro with inflamed OA chondrocytes or synoviocytes, ASCs determined an anti-inflammatory action [10], reflected by a decrease in expression of IL1β, IL6, and CXCL8/IL-8. A similar effect was observed in synoviocytes after administration of ASC-derived extracellular vesicles, which are suggested to be the real effectors in cell-based therapies [58]: after 10 days inflammatory markers are significantly downregulated, with C-C motif chemokine ligand 2 (CCL2) and CCL5 returning to pre-inflammation basal levels.

The results collected in this systematic review about the effects on the inflammatory/catabolic status of the OA joint measured in the SF or serum reported a decrease in levels of lymphocytes and inflammatory biomarkers, indicating strong anti-inflammatory action of ASCs, and the reduction of MMP3, MMP13 and CTX2, which indicates a positive effect on matrix degradation [ε]. The interaction between ASCs with the degenerated/inflamed environment is a crucial factor in understanding their role. SF is a good indicator of the joint's general condition as the content of biomarkers correlates with the inflammatory/pathological state of cartilage and synovial membrane [59]. In a recent article, SF analysis of OA patients detected several cytokines and chemokines linked to inflammation, where IL-6 and IL-8 were the most abundant factors [60]. The secretory profile of ASCs treated with these SF samples revealed more than 50 factors in the cell secretome mainly involved in the organization and homeostasis regulation of the extracellular matrix, the interaction with cells of the immune system, and the regulation of cytokine production, including TNFα, IL-1β, and IL6 receptors [60]. Overall, these results suggest a possible molecular explanation for the effects observed in vivo in most of the studies included in the systematic review.

Results as objective as those reported in animals are difficult to reproduce in humans due to various ethical and practical concerns. Only a few clinical studies attempted to evaluate disease-modifying effects following injection of adipose-derived cell-based therapies in OA patients, including MFAT, SVF, and ASCs [61–64]. In this light, the analysis of the preclinical literature can help identify effects and mechanisms of action, as well as more promising strategies (e.g., number of cells, number of injections, preculturing, etc.). However, a critical issue is underlined by this systematic review. Almost all the preclinical studies (65 out of 71) focused on the use of culture-expanded ASCs, while only 6 studies reported the use of products prepared at the point of care (SVF or MFAT). Interestingly, this is in countertrend if compared to clinical trials where the majority reported results of products prepared at the point of care from autologous adipose tissue [65]. Orthobiologics prepared at the point of care by minimal manipulation are easier to use, more cost-effective than culture-expanded cells, and more accessible in the clinical routine from the regulatory perspective.

The current systematic review also highlights the effects of these products in relation to the protocol of application, dosage, processing methods (cultured ASCs or not) and in comparison to other injective therapies. While providing some interesting preliminary indications, this analysis did not result in sufficient information to propose strong recommendations, with the findings collected on these particular aspects scarce and often controversial. Similar to clinical trials [66], preclinical studies also suffer from inconsistencies in results due to excessive heterogeneity of methods relating to the use of both cultured ASCs and point of care products. In fact, no consensus regarding methods has been achieved so far, therefore no gold standard has been identified. However, from the aspects evaluated in this review, the augmentation of adipose-derived cell-based products with other therapies or by cellular pre-treatments provided more consistent results. A synergistic effect was observed in inhibiting cartilage degeneration and increasing the synthesis of extracellular matrix, the proliferation of chondrocytes, and the induction of anti-inflammatory effects when ASCs (both expanded and SVF) were associated with hyaluronic acid [ο] and when expanded ASCs were associated with PRP [46, 47], respectively. A few clinical studies showed the potential benefit of the combined use of SVF and PRP [67–70], so it would be interesting to conduct targeted pre-clinical and clinical studies investigating the combined use of cell-based products and PRP to further understand the possible synergistic effect of these orthobiologic agents.

The current systematic review has several limitations. The major one is the high heterogeneity found among the studies. In fact, different conditions related to the type of animal models, different follow-ups and measures, and different procedures are reported, which make the analysis of the outcomes more complex and the comparison of the effects of the therapies less reliable. The heterogeneity in methods, formulations, and results highlights the complexity of this area that should be also attributed to their recent introduction, and further highlights the need for achieving and setting clearer recommendations that are not yet available to date. Adhering to specific guidelines, for example using the SYRCLE’s tool to reduce the risk of bias when planning a study, would improve the quality and reliability of the results and increase the homogeneity of the studies, thus favouring more in-depth literature analyses. Another limitation of the study is that the pathophysiology of OA in animals and also the cartilage composition—especially in the smaller ones—as well as their response to treatment do not exactly represent the human equivalent conditions. Nevertheless, the biological effects of adipose tissue derived cell-based injective therapies seen in animal models may be considered as an indicator for potential processes and effects when orthobiologics are used in humans. Animal OA models could still be able to partially reproduce the effects of orthobiologics in human OA joints. Moreover, animal experiments also have the advantage when compared to human trials in terms of no placebo effect influence on the study findings, which makes the clinical improvement documented in this systematic review meaningful, with 78% of the clinical studies documenting a significant clinical improvement. Interestingly, imaging was the method which documented the lowest effect of adipose-derived cell-based therapy, suggesting that this approach is limited in its ability to detect disease-modifying effects in comparison to others utilised. MRI and radiographic evaluations are currently the most common option applied in the clinical setting and showed the lowest ability in detecting the effects of the applied treatments. This confirms the limits of the imaging evaluations already documented in the clinical practice, where there is a poor correlation between clinical outcome and imaging findings [71]. In this light, the limits of imaging techniques should be considered with caution to draw conclusions on treatment effectiveness in the clinical practice, as more disease-modifying effects have been found in the literature using other methods rather than MRI and radiographs, as underlined by the tissue and biomarker analysis in the animal models. These preclinical results could also serve as a useful comparison of different approaches to help identifying the most suitable cell-based strategy to be translated to humans for the treatment of OA.

Overall, this review provided a systematic view of the available literature about the disease-modifying effects of adipose-based orthobiologics in the treatment of OA in the preclinical setting, and therefore without being affected by the bias typical of the clinical literature. The information collected, in addition to contributing to the transition to the clinical setting of these therapeutic solutions, represents an important tool to understand their effects and to guide further research to optimize their use based on the clinical need.

## Conclusions

Current preclinical models offer the opportunity to document consistent evidence of disease-modifying effects of adipose-derived cell-based therapies for the treatment of OA. Positive results have been observed at both the cartilage and synovial level, as well as in the analysis of biomarkers, clinical, and imaging results in most of the evaluated animals. The risk of bias and overall low quality of the published studies highlight the need for further targeted research to provide recommendations on the optimal methodologies required for a more effective application of adipose-derived cell-based therapies for the treatment of OA.

## References groups

α: [21, 22, 36, 49, 72, 73]

β: [19, 21, 28, 32, 45, 74–76]

γ: [19, 49, 74, 77]

δ: [19, 23, 24, 39]

ε: [28, 44, 78]

ζ: [46, 50, 79, 80]

η: [50, 81–84]

θ: [26, 38, 49]

ι: [23, 28, 30, 79]

κ: [19, 27, 29, 82, 85]

λ: [25, 73, 79, 86–89]

μ: [44, 79, 90–92]

ν: [42, 93]

ξ: [31, 46, 47]

ο: [23, 24, 28, 45, 48–50, 94]

## Supplementary Information

Below is the link to the electronic supplementary material.

Supplementary file1 (DOCX 28 KB)
