# Supplementary material for: Cell-based therapies have disease-modifying effects on osteoarthritis in animal models. A systematic review by the ESSKA Orthobiologic Initiative. Part 1: adipose tissue-derived cell-based injectable therapies
Source: Knee Surg Sports Traumatol Arthrosc. 2022 Sep 14;31(2):641–55. doi: 10.1007/s00167-022-07063-7 (PMC9898370; doi:10.1007/s00167-022-07063-7)
Supplement: Supplementary file 1 — Supplementary file1 (DOCX 28 KB) [file 167_2022_7063_MOESM1_ESM.docx]

**Table 1**. Characteristics of the included studies.

| **Authors Journal Year** | **Animal Model** | **Evaluated  Joint** | **OA Model** | **Treatment Groups** | **ASCs Origin** | **Expanded or Point of Care** | **ASCs Dose** | **Additional  Procedures** | **Injection Protocol** N. injections Injection timing Injected Volume | **Follow-up** | **Results** |
| --- | --- | --- | --- | --- | --- | --- | --- | --- | --- | --- | --- |
| Cheng JH et al. Pharmaceuticals 2021 [23] | 42 Rats | Knee | ACL transection  and medial meniscectomy | Low-dose ASC (n=6) High-dose ASC (n=6) SW therapy (n=6) Low-dose ASC + SW (n=6) High-dose ASC + SW (n=6) OA control (n=6) Healthy control (n=6) | Autologous  (Rat) | Expanded | Low-dose ASC: 1 x 10^6^ High-dose ASC: 2 x 10^6^ | - | Single injection - 100 μL | 3 months | ASCs treatment exhibited a dose-dependent effect and a synergistic action with SW therapy in the protection of the articular cartilage with inflammation. |
| Filardo G et al. KSSTA 2021 [34] | 48 Rabbits | Knee | ACL transection | ASC (n=12) MFAT (n=12) SVF (n=12) OA Control (n=12) | Autologous  (Rabbit) | Expanded and Point of care | ASC: 2 x 10^6^ | - | Single injection - 300 μL | 4 months | All treatment types showed therapeutic potential, with the MFAT showing the most promising results. |
| Hsu GCY et al. J Orthop Res 2021 [25] | 40 Mice | Knee | Destabilized medial meniscus | ASC Pdgfrα^+^ (n=10) ASC Pdgfrβ^+^ (n=10) OA control (n=10) Sham control (n=10) | Allogeneic  (Mouse) | Expanded | 1 x 10^6^ | - | Single injection - - | 2 months | ASCs showed improvement in the OARSI score and the Pdgrfβ^+^ group showed less subchondral sclerosis. |
| Kamada K et al.  J Clin Med  2021 [20] | 66  Mice | Knee | Destabilized medial meniscus | ASC (n=33) OA control (n=33) | Xenogeneic (Human) | Point of care | 2 x 10^4^ | - | Single injection - 6 µL | 2 months | ASCs attenuated OA progression, suppressing the production of cartilage-degrading enzymes and inflammatory cytokines. |
| Keller LE et al. Cartilage 2021 [95] | 8 Horses | Ankle | Focal impact injuries | ASC (n=8 knees) OA control (n=8 knees) | Allogeneic (horse) | Expanded | 2 x 10^7^ | - | Single injection - 3 mL | 6 months | Decreased expression of TIMP2 and NF-kβ and increased expression of CCL5 in joints treated with ASCs. |
| Khonke R et al. Int J Mol Sci 2021 [32] | 28 Rabbits | Temporo-mandibular | Collagenase  injection | ASC (n=7) ASC + HA (n=7) HA (n=7) OA control (n=7) | Xenogeneic  (Human) | Expanded | 1 x 10^6^ | - | Single injection - 150 μL | 2 months | The results suggest that intra-articular injection of ASCs can support articular regeneration in temporo-mandibular OA. |
| Ko JY et al. J Biomed Mater Res A 2021 [37] | 12 Rats | Knee | ACL transection  and medial meniscectomy | ASC (n=4) ASC + spheroid (n=4) OA control (n=4) | Xenogeneic  (Human) | Expanded | 1 x 10^6^ | ASCs + spheroid group: ASCs cultured in 3D cell culture dishes with spheroids | Single injection - 100 μL | 2 months | ASCs spheroids arrested the progression of surgically induced OA better than ASCs alone. |
| Oh J et al. J Orthop Surg Res 2021 [88] | 16 Dogs | Knee | ACL transection | ASC (n=4) PDGF-ASC (n=4) HO-1-ASC (n=4) OA control (n=4) | Allogeneic  (Dog) | Expanded | 2 x 10^7^ | Lentiviral transduction of PDGF or HO-1 | Single injection - 1 mL | 4 months | ASCs yielded improvement in clinal outcomes, with the PDGF transfected ASCs exhibiting the best outcomes. |
| Veronesi F et al. Int Orthop 2021 [22] | 12 Sheep | Knee | Meniscectomy | ASC (n=6 knees) SVF (n=6 knees) AEC-MSC (n=6 knees) OA control (n=6 knees) | Autologous  (Sheep)  + Xenogeneic  (Human) | Expanded | 2.5 x 10^6^ | - | Single injection - 1 mL | 3 months | All treatments provided better biochemical, gross and biomechanical results compared to the control group. AEC and SVF showed better results. |
| Zhang R et al. Am J Transl Res 2021 [76] | 30 Rabbits | Knee | ACL transection  and medial meniscectomy | ASC (n=12) OA control (n=12) Healthy control (n=6) | Allogeneic  (Rabbit) | Expanded | 6 x 10^6^ | - | Single injection - 200 µL | 2 months | Intra-articular injection of ASCs evidently ameliorated articular cartilage damage and retarded OA progression. |
| Ahmad MR et al. Regen Med 2020 [46] | 50 Rats | Knee | ACL transection  and medial meniscectomy | ASC (n=10) PRP (n=10) ASC+PRP (n=10) OA control (n=10) Healthy control (n=10) | Allogeneic  (Rat) | Expanded | 1 x 10^6^ | Preconditioned  with Vitamin E | Single injection - 100 μL | 1 month | ASCs reduced inflammatory biomarkers, improved the proteoglycan content and gene expression of cartilage. ASCs improved the effect of PRP. |
| An XY et al. Aging and Disease 2020 [50] | 35 Rabbits | Knee | Joint  immobilization | ASC (n=7) Acupotomy (n=7) ASC+Acupotomy (n=7) OA control (n=7) Healthy control (n=7) | Xenogeneic  (Human) | Expanded | 1 x 10^5^ | - | Single injection - 2 mL | 1 week | ASCs provided positive results in knee OA rabbits, resulting in better findings in the combination therapy with acupotomy. |
| Delco L et al. Am J Sports Med 2020 [18] | 8 Horses | Ankle | Impact-induced articular injury | ASC (n=8 knees) OA control (n=8 knees) | Allogeneic  (Horse) | Expanded | 2 x 10^7^ | ASC with high surface expression of integrin α10β1 | Single injection - 3mL | 6 months | ASCs resulted in less cartilage fibrillation and less subchondral bone sclerosis at 6 months after articular injury. |
| Hsu CC et al. Int J Mol Sci 2020 [24] | 42 Rats | Knee | ACL transection  and medial meniscectomy | ASC (n=6) Wharton’s jelly MSCs (n=6) ASC + ESWT (n=6) Wharton’s jelly MSCs + SW therapy (n=6) SW therapy (n=6) OA control (n=6) Sham control (n=6) | ASC: Autologous  (Rat)  Wharton’s jelly: Xenogeneic  (Human) | Expanded | ASC: 1 x 10^6^ Wharton’s jelly MSC: 1 x 10^6^ | - | Single injection - 200 μL | 4 months | Combined SW therapy with ASCs was found to be effective for the treatment of early knee OA in rats. |
| Kim JH et al. J Vet Clin 2020 [45] | 50 Rabbits | Knee | ACL transection  and medial meniscectomy | ASC (n=6) Carboxymethyl Chitosan (n=6) ASC + Carboxymethyl Chitosan (n=6) HA (n=6) OA Control (n=6) | Allogeneic  (Rabbit) | Expanded | 2 x 10^6^ | - | Three injections - 0.3 mL | 2 months | ASCs, Carboxymethyl Chitosan, or their combination treatment favorably ameliorates the surgically-induced OA in rabbits. |
| Lee JC etl al. J Anat Soc India 2020 [96] | -  Rats | Knee | MIA  injection | ASC ASC exosome OA control Healthy control | Xenogeneic  (Human) | Expanded | 2 x 10^6^ | - | -  -  - | - | Microarray analysis showed an increase of expression of several genes in the ASCs and exosome groups compared with controls. |
| Maki CB et al. Front Vet Sci 2020 [82] | 20 Dogs | Hip | Natural  occurring | Low-dose ASC (n=5) Mid-dose ASC (n=6) High-dose ASC (n=5) OA control (n=4) | Allogeneic  (Dog) | Expanded | Low-dose ASC: 5 x 10^6^  Mid-dose ASC: 25 x 10^6^  High-dose ASC: 50 x 10^6^ | - | Single injection - 0.6 mL | 3 months | ASCs was well-tolerated and improved the lameness score and increased mobility of dogs suffering from hip OA. |
| Song SY et al. Adv Healthc Mater 2020 [38] | 18 Rats | Knee | ACL transection  and medial meniscectomy | ASC (n=3)  IL-4 ASC (n=3)  ASC spheroid (n=3) IL-4 ASC spheroid (n=3) OA control (n=3) Healthy control (n=3) | Allogeneic  (Rat) | Expanded | 5 x 10^5^ | IL4 transfection and/or ASC spheroid creation (300 cells) | Single injection - 50 µL | 2 months | IL-4 ASCs spheroids showed better cartilage protection and pain relief than conventional naïve ASCs. |
| Takagi T et al. BMC Musculoskelet Disord 2020 [77] | 24 Rabbits | Knee | ACL transection | ASC (n=24 knees) OA control (n=24 knees) | Autologous  (Rabbit) | Expanded | 1 x 10^6^ | Fabrication of ASC sheets | Multiple injections 1-week intervals 1 mL | 3 months | Periodic injections of ASCs sheets attenuated OA progression in an experimental rabbit model. |
| Wang Z et al. Stem Cell Investig 2020 [97] | 24 Rats | Knee | Medial  meniscectomy | ASC (n=12) OA control (n=12) | Xenogeneic  (Human) | Expanded | 1.25 x 10^6^ | - | Single injection - 50 µL | 1 month | Intra-articular injection of ASCs alleviates OA-induced joint pain. |
| Wits MI et al. Genet Mol Biol 2020 [84] | 12 Dogs | Hip | Natural  occurring | ASC (n=4) ASC + HA (n=4) OA control (n=4) | Allogeneic  (Dog) | Expanded | 5 x 10^6^ | - | Single injection - 0.5 mL | 3 months | Intra-articular cellular treatment alleviated OA-induced joint pain and improved lameness in dogs. |
| Desando et al. Int J Mol Sci 2019 [19] | 18 Rabbits | Knee | ACL transection | ASC (n=6) MFAT (n=6) SVF (n=6) | Allogeneic  (Rabbit) | Expanded and Point of care | ASC: 2 x 10^6^ | Cell labeling with PKH26 Red Fluorescent Cell Linker | Single injection - 300 µ | 1 month | All types of cells yielded repair response in joint tissue. |
| Dubey NK et al. Aging Dis 2019 [74] | 17 Mice | Knee | Intraperitoneal streptozotocin injection | ASC (n=6) Saline (n=6) Healthy control (n=5) | Allogeneic  (Mouse) | Expanded | 1 x 10^6^ | - | Single injection - 0.5mL | 1 month | The administered ASCs halted the OA characteristics in diabetic mice. |
| Ko JY et al. Tissue Eng Part A 2019 [33] | 30 Goats | Knee | Medial  meniscectomy | ASC (n=6) low-dose ASC^SOX^ (n=6) mid-dose ASC^SOX^ (n=6) high-dose ASC^SOX^ (n=6) OA control (n=6) | Xenogeneic  (Human) | Expanded | ASC: 0.6 x 10^7^ Low-dose ASC^SOX^:  0.18 x 10^7^ Mid-dose ASC^SOX^:  0.6 x 10^7^ High-dose ASC^SOX^:  1.8 x 10^7^ | ASC^SOX^: Construction of SOX 6,9 plasmid and transferring it to the ASCs | Single injection - - | 5 months | ASCs (especially at a mid-dose) effectively arrested OA progression in surgically-induced OA. |
| Kuroda K et al. J Orthop Res 2019 [72] | 24 Rabbits | Knee | ACL transection | ASC (n=12) OA control (n=12) | Autologous  (Rabbit) | Point of care | 1 x 10^5^ | - | Single injection - 1 mL | 3 months | ASCs inhibited cartilage degeneration progression in a rabbit OA model. |
| Li JR et al. Exp Ther Med 2019 [98] | 40 Rats | Knee | Collagenase  injections | ASC (n=20) OA control (n=20) | Allogeneic  (Rat) | Expanded | - | - | -  -  - | 1 month | ASCs improve knee joint evaluation and gene expressions in the rat OA model. |
| Sakamoto et al. BMC Musculoskelet Disord 2019 [40] | 90 Rats | Knee | MIA injection | ASC early (n=30) ASC late (n=20) OA control (n=40) | Allogeneic  (Rat) | Expanded | 1 x 10^6^ | - | Single injection - - | 1 month | ASCs prevented degenerative changes in the early injection group but had little effect in the late injection group. |
| Shin K et al. World J Stem Cells 2019 [28] | 36 Rabbits | Knee | ACL transection | High-dose ASC (n=6) Low-dose ASC (n=6) Low-dose ASC + TSP2 (n=6) TSP2 (n=6) OA control (n=6) Sham control (n=6) | Xenogeneic  (Human) | Expanded | Low-dose ASC:  1.7 x 10^6^ High-dose ASC:  1.7 x 10^7^ | - | Single injection - 0.5 mL | 2 months | Cellular therapies attenuated OA progression in a rabbit model, and the combination therapy with ASCs and TSP2 exerted synergistic efficacy. |
| van Dalen SCM et al. Front Immunol 2019 [17] | 66  Mice | Knee | Collagenase  injections | Low-dose ASC (n=22) High-dose ASC (n=22) OA Control (n=22) | Allogeneic  (Mouse)   + Xenogeneic  (Human) | Expanded | Low-dose ASC: 2 x 10^4^ High-dose ASC: 1 x 10^5^ | - | Single injection - 6 µL | 1 month | ASCs injection resulted in comparable numbers of PMNs which clustered around ASCs in close interaction with the synovial lining. |
| Xie M et al. RSC Advances 2019 [99] | -  Rats | Knee | Medial  meniscectomy | ASC Oa control | Allogeneic  (Rat) | Expanded | 2 x 10^6^ | - | Single injection - 100 μL | 2 months | ASCs provided positive results on cartilage healing. |
| Zhou J et al. J Cell Biochem 2019 [78] | -  Rats | Knee | Modified  Hulth method | ASC OA control | Allogeneic  (Rat) | Expanded | 2 x 10^6^ | - | Eight injections two times a week 50 µL | 1 month | ASCs reduced the secretion of proinflammatory cytokines. |
| Choi S et al.  Stem Cells Int 2018 [36] | 11 Rabbits | Knee | ACL transection | ASC (n=5 knee)  Microencapsulated ASC (n=5 knees) Alginate beads (n=6 knees) OA control (n=6 knees) | Allogeneic  (Rabbit) | Expanded | 1 x 10^7^ | Microencapsulation | Three injections  1-week interval 1 mL | 2 months | Microencapsulated ASCs slowed the progression of OA and decreased its extent, more so than did free ASCs. |
| Feng et al. Tissue Eng Part A 2018 [29] | 24 Sheep | Knee | ACL transection  and medial meniscectomy | Low-dose ASC + HA(n=6)  High-dose ASC + HA (n=6) HA (n=6) Saline (n=6) | Allogeneic  (Sheep) | Expanded | Low-dose ASC: 1 x 10^7^ High-dose ASC: 5 x 10^7^ | - | Two injections 3-week interval 5mL | 4 months | ASCs combined with HA could effectively block OA development and promote cartilage regeneration. |
| Jacer S et al. Adv Pharm Bull  2018 [48] | 15 Rats | Knee | Collagenase  injection | ASC Chondral cell suspension ASCs + Chondral cell suspension OA control Healthy control | Allogeneic  (Rat) | Expanded | 1 x 10^7^ | - | Single injection - 20 μL | 3 months | ASCs and chondral cell suspension provided positive results compared to OA controls. |
| Lee JC et al. Genes Genomics 2018 [73] | -  Rats | Knee | MIA  injection | ASC OA control Healthy control | Xenogeneic  (Human) | Expanded | 3.0 x 10^6^ | - | Single injection - 200 μL | 1 month | Changes in gene expression in the ASCs group may indicate possible repair pathways. |
| Lee SY et al. Front Immunol 2018 [26] | 15 Rats | Knee | MIA  injection | ASC (n=3) OA-ASC (n=3) iSTAT3 OA-ASC (n=3) OA control (n=3) Healthy control (n=3) | Xenogeneic  (Human) | Expanded | 3 x 10^5^ | STAT3 OA-ASCs were prepared by treatment MSCs with 10 μM STA21 | Two injections 4-week interval 50 μL | 2 weeks | STAT3 inhibition of ASCs may optimize their therapeutic activity in attenuating OA progression. |
| Liu XW et al. Exp Ther Med 2018 [87] | 40 Mice | Knee | ACL transection | ASC (n=8) ASC + BMP9 (n=8) ASC + BMP9 + LY411575 (n=8) OA control (n=8) Healthy control (n=8) | Unspecified | Expanded | 1 x 10^7^ | BMP9 transfection | Four injections 1-week interval 10 μL | 1 month | ASCs contributed to cartilage repair affected knee joint in mice. |
| Lv XT et al. Cell Transplantation 2018 [30] | 30  Sheep | Knee | ACL transection  and medial meniscectomy | Low-dose ASC + HA (n=6) High-dose ASC + HA (n=6) SVF + HA (n=6) HA (n=6) OA control (n=6) | Autologous  (Sheep) | Expanded and Point of care | Low-dose ASC: 1 x 10^7^ High-dose ASC: 5 x 10^7^ SVF: 5 x 10^7^ MNC cells | 2.5 ml of HA added MSCs cryopreserved for the 2nd injection | Two injections 3-week interval 5mL | 6 months | ASCs + HA result in better efficacy than SVF+HA in blocking OA progression. ASCs + HA provided better results compared to HA alone. |
| Marinas-Pardo L et al. Stem Cells Dev 2018 [83] | 72 Horses | Distal inter-phalangeal or metatarso-phalangeal joints | Natural  occurring | ASC (n=36) OA control (n=36) | Allogeneic  (Horse) | Expanded | 1 x 10^7^ | - | Three injections 2-week interval 2 mL | 3 months | ASCs reduced lameness induced by OA for an extended period of 90 days. |
| Stancker TG et al. Lasers Med Sci 2018 [94] | 50  Rats | Knee | Papain  injections | ASC (n=10) ASC + PBMT (n=10) PBMT (n=10) OA control (n=10) Sham control (n=10) | Allogeneic  (Rat) | Expanded | 1 x 10^7^ | ASC were irradiated after implantation | Single injection - 60 µL | 1 week | The intra-articular injection of ASCs and PBMT prevented joint degeneration. |
| Sun Q et al. Biotech Histochem 2018 [16] | 30 Rabbits | Knee | Medial  meniscectomy | ASC (n=10) ASC + tgf-3- microspheres (n=10) OA control (n=10) | Xenogeneic  (Human) | Expanded | 2 x 10^6^ | TGF-β3 microspheres | Single injection - 0.2 mL | 3 months | ASCs inhibited OA progression. TGF-β3 microspheres can produce synergistic effect. |
| Ude CC et al. Exp Gerontol 2018 [91] | 18 Sheep | Knee | ACL transection  and medial meniscectomy | ASC (n=6) BMSC (n=6) OA control (n=6) | Autologous  (Sheep) | Expanded | 2 x 10^7^ | Cells were induced to chondrogenic lineage | Single injection - 5 mL | 12 months | Each of the treated groups had significantly improved cartilage scores compare to the OA controls. |
| Mei L et al. Biochem Biophys Res Commun 2017 [49] | 64  Rats | Knee | ACL transection | ASC ASC + XG XG OA control Sham control | Allogeneic  (Rat) | Expanded | 1 x 10^6^ | XG added in the ASC+XG group | Single injection - 60 µL | 2 months | ASCs attenuated OA progression in a rat model. The combined use of XG improved results vs ASCs  monotherapy. |
| Mei L et al. PLoS One 2017 [21] | 60 Rats | Knee | ACL transection | ASC (n=20) OA control (n=20) Healthy control (n=20) | Allogeneic  (Rat) | Expanded | 1 x 10^6^ | - | Single injection - 60 µL | 3 months | ASCs attenuated cartilage degeneration without inducing any adverse effects. |
| Munoz-Criado I et al. Stem Cells Int 2017 [31] | 24 Mice | Knee | Collagenase  injection | ASC (n=6) SVF (n=6) PRP (n=6) OA (n=6) | Xenogeneic  (Human) | Expanded | 1 x 10^5^ | - | Single injection - 6 µL | 1 month | Suprapatellar fat pad offers a proper ASCs source for cartilage regeneration. |
| Parrilli A etl a. Biotech Histochem 2017 [27] | 12  Rabbits | Knee | ACL transection | Low-dose ASC (n=4) High-dose ASC (n=4) OA control (n=4) | Autologous  (Rabbit) | Expanded | Low-dose ASC: 2 x 10^6^ High-dose ASC: 6 x 10^6^ | - | Single injection - - | 6 months | ASCs have contribution to bone turnover in OA model. |
| Riester SM et al. Stem Cells Transl Med 2017 [85] | 18 Rabbits | Knee | Medial  meniscectomy | Low-dose ASC (n=6) High-dose ASC (n=6) OA control (n=6) | Xenogeneic  (Human) | Expanded | Low-dose ASC: 2 x 10^6^ High-dose ASC: 6 x 10^6^ | - | Single injection - 1 mL | 2 months | No cartilage regeneration or clinical response were seen in the treatment group. |
| Tang Y et al. J Cell Mol Med 2017 [43] | 40 Rats | Knee | MCL tear + Medial meniscal tear | Subcutaneous ASC (n=10) Visceral ASC (n=10) OA control (n=10) Sham control (n=10) | Xenogeneic  (Mouse) | Expanded | 4 x 10^6^ | - | Single injection - 6 μL | 2 months | Subcutaneous ASCs has better healing effect than visceral ASCs for knee OA treatment. |
| Harman R et al. Front Vet Sci 2016 [100] | 74 Dogs | Elbow, Knee, Hip, Shoulder | Natural  occurring | ASC (n=38) Saline (n=36) | Allogeneic  (Dog) | Expanded | 12 x 10^6^ | - | Single injection - 0.7 mL | 2 months | ASCs treatment was shown to be efficacious compared to placebo. |
| Hermeto LC et al. Genet Mol Res 2016 [42] | 24 Rabbits | Knee | Collagenase  injections | undifferentiated ASC+PRP (n=6) differentiated ASC + PRP (n=6) PRP (n=6) Oa control (n=6) | Autologous  (Rabbit) | Expanded | 4 x 10^6^ | Cells were induced to chondrogenic lineage | Single injection - - | 2 months | Combination therapy of ASCs and PRP is beneficial, while no differences were found between differentiated and undifferentiated ASCs. |
| Latief N et al. Cell Biol Int 2016 [41] | 48 Rats | Knee | ACL transection  and medial meniscectomy | ASC (n=12) Differentiated ASCs (n=12) OA control (n=12) Healthy control (n=12) | Unspecified | Expanded | 1 x 10^5^ cells | Cells were induced to chondrogenic lineage | Single injection - 100 μL | 1 month | ASCs and differentiated- ASCs improved cartilage damage in the rat OA model. Differentiated cells were more effective than regular ASCs. |
| Li M et al. Stem Cell Res Ther 2016 [75] | 18 Rats | Knee | Medial  meniscectomy | ASC (n=6) OA control (n=6) Healthy control (n=6) | Xenogeneic (Human) | Expanded | 2.5 x 10^6^ | ASC labeling with DiD fluorescent dye | Single injection - 100 μL | 2 months | ASCs exerted therapeutic effect on the knee joint. |
| Maumus M et al. Front Immunol 2016 [79] | 60 Mice | Knee | Collagenase  injections | Low-dose ASC (n=10) High-dose ASC (n=10) Low-dose IFNγ-ASC (n=10) High-dose IFNγ-ASC (n=10) BMSC (n=10) OA control (n=10) | ASC: Xenogeneic  (Horse)  BMSCs: Allogeneic  (Mouse) | Expanded | Low-dose ASC: 2 x 10^4^ High-dose ASC: 2 x 10^5^ BMSCs: 2 x 10^5^ | Primed or not with IFNγ (100 ng/mL for 24 h) | Single injection - 8 µL | 1 month | IFNγ-pretreatment of ASCs greatly improved their therapeutic efficacy in terms of chondroprotective functions for OA treatment. |
| Mohoric L et al. Slov Vet Res 2016 [101] | 10 Dogs | Knee | Natural  occurring | ASC (n=10 knees) OA control (n=10 knees) | Autologous  (Dog) | Expanded | 2-3 x 10^6^ | - | Single injection - 1 mL | 12 months | ASCs therapy seemed to slow down or even stopped degenerative processes. |
| Schwabe K et al. Arthritis Rheumatol 2016 [89] | 24 Mice | Knee | Collagenase  injections | ASC (n=6) Fra-1 ASC (n=6) OA control (n=6)  Healthy control (n=6) | Allogeneic  (Mouse) | Expanded | 2 x 10^4^ | Overexpression of Fra-1 | Single injection - 6 µL | 1 month | Fra-1 ASCs improve the healing capabilities of ASCs in the OA model. |
| Yun S et al. J Orthop Surg Res 2016 [47] | 24 Dogs | Knee | ACL transection | ASC (n=6) ASC +PRP (n=6) PRP (n=6) OA Control (n=6) | Allogeneic  (Dog) | Expanded | 1 x 10^7^ | - | Four injections 1-week interval 1 mL | 2 months | ASCs and/or PRP ameliorated the degeneration of articular cartilage, with better results in the combination therapy. |
| Kuroda K et al. BMC Musculoskelet Disord 2015 [102] | 12 Rabbits | Knee | ACL transection | ASC + HA (n=12 knees) HA (n=12 knees) | Allogeneic  (Rabbit) | Expanded | 2 x 10^6^ | - | Three injections 1-week interval 200 μL | 3 months | ASCs and HA injections had a higher inhibiting effect on cartilage degeneration compared to HA alone. |
| Toupet K et al. PLoS One 2015 [80] | 20 Mice | Knee | Collagenase  injection | ASC (n=10) OA control (n=10) | Xenogeneic  (Human) | Expanded | 2.5 x 10^5^ | - | Single injection - 7 µL | 1 month | Intra-articular administration of ASCs inhibited OA progression. |
| Ude CC et al.  Osteoarthritis Cartilage 2015 [90] | 9 Sheep | Knee | ACL transection  and medial meniscectomy | ASC (n=3) BMSC (n=3) OA Control (n=3) | Autologous  (Sheep) | Expanded | 2 x 10^7^ | Cells were induced to chondrogenic lineage | Single injection - 5 mL | 7 months | BMSCs and ASCs achieved similar gross and clinical results in the sheep OA model. |
| Wang W et al. Int J Mol Sci 2015 [103] | 12 Rabbits | Knee | ACL transection  and medial meniscectomy | ASC + HA (n=6 knees) HA (n=6 knees) Healthy control (n=12 knees) | Xenogeneic (Human) | Expanded | 2.5 x 10^6^ | - | Three injections - 0.15 mL | 4 months | ASCs + HA performed better results than HA in cartilage healing in the OA model. |
| Cuervo B et al. Int J Mol Sci 2014 [93] | 35 Dogs | Hip | Natural  occurring | ASC (n=18) PGRF (n=17) | Autologous  (Dog) | Expanded | 3 x 10^7^ | - | Single injection - 2 mL | 6 months | Compared to PRGF, ASCs showed better results at 6 months. |
| Nicpon J et al. Kafkas Uni Vet Fak Der 2014 [104] | 12 Dogs | Elbow | Natural  occurring | ASC (n=6) NSAIDs (n=6) | Autologous  (Dog) | Expanded | 1.5x10^6^ | - | Single injection - 1 mL | 6 months | ASCs improved the clinical picture and the dogs’ condition. |
| Schelbergen RF et al. Osteoarthritis Cartilage 2014 [105] | Study I 64 Mice | Knee | Collagenase  injection | ASC (n=32) OA control (n=32) | Unspecified  (Mouse) | Expanded | 2 x 10^4^ | - | Single injection - 6 µL | 1 month | The results suggest that the healing capacity of ASCs is related to synovitis. |
|  | Study II 36 Mice | Knee | Destabilized medial meniscus | ASC (n=18) OA control (n=18) | Unspecified  (Mouse) | Expanded | 2 x 10^4^ | - | Single injection - 6 µL | 1 month |  |
| Ude CC et al. PLoS One 2014 [92] | 18 Sheep | Knee | ACL transection  and medial meniscectomy | ASC (n=6) BMSC (n=6) OA control (n=6) | Autologous  (Sheep) | Expanded | 2 x 10^7^ | Cells were induced to chondrogenic lineage | Single injection - 5 mL | 1 month | There was no difference of ICRS score between ASCs and BMSCs treatment. Both groups achieved significantly better results vs control group. |
| Desando et al. Arthritis Res Ther 2013 [19] | 93 Rabbits | Knee | ACL transection | Low-dose ASC (n=24) High-dose ASC (n=24) 4% RSA (n=24) OA control (n=9) Biodistribution (n=9) Sham control (n=3) | Autologous  (Rabbit) | Expanded | Low-dose ASC: 2 x 10^6^ High-dose ASC: 6 x 10^6^ | - | Single injection - 1mL | 1 month | ASCs showed healing effect on the menisci and cartilage, and an inhibition of OA progressions in the synovial membrane. |
| Lee JM et al. Biomaterials 2012 [86] | 9 Rats | Knee | ACL transection | ASC (n=3) SOX-ASC (n=3) OA control (n=3) | Autologous  (Rat) | Expanded | 1 x 10^6^ | SOX transduction | Single injection - 0.2 mL | 2 months | SOX trio-co-transduced ASCs arrested the progression of surgically-induced osteoarthritis in a rat model. |
| Ter Huurne M et al.  Arthritis Rheum 2012 [39] | -  Mice | Knee | Collagenase  injection | Early-ASC  (7 days after induction) Late-ASC  (14 days after induction) OA control | Unspecified  (Mouse) | Expanded | 2 x 10^4^ | - | Single injection - 6 µL | 1 month | ASCs when given in early OA inhibit synovial lining thickening and protect against joint destruction. |
| Toghraie FS et al. Arch Iran Med 2012 [106] | 28 Rabbits | Knee | ACL transection | ASC (n=10) OA control (n=10) Sham control (n=8) | Allogeneic  (Rabbit) | Expanded | 1 x 10^6^ | - | Single injection - 1 mL | 5 months | Intra-articular ASCs improved histological OA score. |
| Toghraie FS et al. Knee 2011 [107] | 20 Rabbits | knee | ACL transection | ASC (n=10) OA control (n=10) Sham control (n=8) | Allogeneic  (Rabbit) | Expanded | 1x 10^6^ cells | - | Single injection - 1 mL | 5 months | ASCs can reduce the development of OA lesions in a rabbit model. |
| Frisbie DD et al. J Orthop Res 2009 [44] | 24 Horses | Knee | Osteochondral  defects | SVF (n=8) BMSC (n=8) Placebo (n=8) | Allogeneic  (Horse) | Point of care | SVF: 16.3 x 10^6^ BMSC: 0.66 x 10^6^ | - | Single injection - 2mL | 2 months | No significant ASCs effects were observed in this OA model. |
| Black LL et al. Vet Ther 2007 [81] | 21 Dogs | Hip | Natural  occurring | ASC OA Control | Autologous  (Dog) | Expanded | 5 x 10^6^ | - | Single injection - 0.6 mL | 3 months | ASCs achieved better clinical results when compared with placebo in dogs with hip OA. |

ACL, anterior cruciate ligament; ASC, adipose-derived MSC; BMP, bone morphogenetic protein; BMSC, bone marrow-derived MSC; IFNγ, interferon-gamma; HA, hyaluronic acid; MCL, medial collateral ligament; MFAT, micro-fragmented adipose tissue; MIA, monosodium iodoacetate; MNC, mononuclear cell; MSC, mesenchymal stromal cell; NSAIDs, non-steroidal anti-inflammatory drugs; OA, osteoarthritis; PBMT, photobiomudulation therapy; PMN, polymorphonuclear cells; PRGF, plasma rich in growth factors; PRP, platelet rich plasma; RSA, rabbit serum albumin; SVF, stromal vascular fraction; SW, shockwave; TSP2, thrombospondin 2; XG, xanthan gum.
